# Supplementary material for: Phosphonic Acid Analogues of Phenylglycine as Inhibitors of Aminopeptidases: Comparison of Porcine Aminopeptidase N, Bovine Leucine Aminopeptidase, Tomato Acidic Leucine Aminopeptidase and Aminopeptidase from Barley Seeds
Source: Pharmaceuticals (Basel). 2019 Sep 17;12(3):139. doi: 10.3390/ph12030139 (PMC6789573; doi:10.3390/ph12030139)
Supplement: Supplementary file 1 [file pharmaceuticals-12-00139-s001.pdf]

pharmaceuticals

Phosphonic acid analogues of phenylglycine as inhibitors of aminopeptidases: Comparison of porcine aminopeptidase N, bovine leucine aminopeptidase and aminopeptidase from barley seeds

Weronika Wanat [1], Michał Talma [1], Małgorzata Pawełczak[2], and Paweł Kafarski *[1]

[1] Department of Bioorganic Chemistry, Wrocław University of Science and Technology, WybrzeżeWyspiańskiego 27, 50-370 Wrocław, Poland;
[2] Faculty of Chemistry, University of Opole, ul. Oleska 48, 45-052, Opole, Poland
* Correspondence: pawel.kafarski@pwr.edu.pl; Tel.: +48 71 320 36 82

Supporting Information

Tables indicating the interactions between phosphonic acid analogues of phenylglycine and porcine aminopeptidase, bovine lens leucine aminopeptidase and tomato acidic leucine aminopeptidase

Table S1. *Interactions of both isomers of compounds* **1-7** *with amino acids of porcine aminopeptidase (pAPN) taken for refinement during the IFD.*

**All**	**1R**	**1S**	**2R**	**2S**	**3R**	**3S**	**4R**	**4S**	**5R**	**5S**	**6R**	**6S**	**7R**	**7S**
**Gln206**					Gln206				Gln206				Gln206	
**Gln208**		Gln208			Gln208				Gln208				Gln208	
**Phe344**		Phe344												
**Asn345**						Asn345								
**Ala346**	Ala346	Ala346	Ala346	Ala346	Ala346	Ala346	Ala346	Ala346	Ala346	Ala346	Ala346	Ala346	Ala346	Ala346
**Gly347**	Gly347	Gly347	Gly347	Gly347	Gly347	Gly347	Gly347	Gly347	Gly347	Gly347	Gly347	Gly347	Gly347	Gly347
**Ala348**	Ala348	Ala348	Ala348	Ala348	Ala348	Ala348	Ala348	Ala348	Ala348	Ala348	Ala348	Ala348	Ala348	Ala348
**Met349**	Met349	Met349	Met349	Met349	Met349	Met349	Met349	Met349	Met349	Met349	Met349	Met349	Met349	Met349
**Glu350**	Glu350	Glu350	Glu350	Glu350	Glu350	Glu350	Glu350	Glu350	Glu350	Glu350	Glu350	Glu350	Glu350	Glu350
**Tyr357**	Tyr357	Tyr357			Tyr357		Tyr357		Tyr357				Tyr357	
**Arg358**				Arg358	Arg358	Arg358	Arg358	Arg358	Arg358	Arg358		Arg358	Arg358	Arg358
**Asn360**														
**Ala361**					Ala361		Ala361	Ala361	Ala361				Ala361	Ala361
**Arg376**	Arg376		Arg376	Arg376	Arg376	Arg376	Arg376	Arg376	Arg376	Arg376	Arg376	Arg376	Arg376	Arg376
**Thr379**	Thr379	Thr379	Thr379								Thr379			
**Val380**	Val380	Val380	Val380	Val380	Val380	Val380	Val380	Val380	Val380	Val380	Val380	Val380	Val380	Val380
**Ile381**														
**His383**	His383	His383	His383	His383	His383	His383	His383	His383	His383	His383	His383	His383	His383	His383
**Glu384**	Glu384	Glu384	Glu384	Glu384	Glu384	Glu384	Glu384	Glu384	Glu384	Glu384	Glu384	Glu384	Glu384	Glu384
**His387**	His387	His387	His387	His387	His387	His387	His387	His387	His387	His387	His387	His387	His387	His387
**Glu406**	Glu406	Glu406	Glu406	Glu406	Glu406	Glu406	Glu406	Glu406	Glu406	Glu406	Glu406	Glu406	Glu406	Glu406
**Ala409**							Ala409	Ala409		Ala409	Ala409	Ala409		Ala409
**Ser410**	Ser410		Ser410	Ser410		Ser410		Ser410		Ser410	Ser410	Ser410		Ser410
**Glu413**	Glu413	Glu413	Glu413	Glu413		Glu413	Glu413	Glu413		Glu413	Glu413	Glu413		Glu413
**Tyr414**														
**Gly433**														
**Asp434**											Asp434			
**Arg437**														
**Phe467**	Phe467	Phe467	Phe467	Phe467	Phe467	Phe467			Phe467	Phe467			Phe467	
**Tyr472**	Tyr472	Tyr472	Tyr472	Tyr472	Tyr472	Tyr472	Tyr472	Tyr472	Tyr472	Tyr472	Tyr472	Tyr472	Tyr472	Tyr472
**Ser473**											Ser473			
**Zn1024**	Zn1024	Zn1024	Zn1024	Zn1024	Zn1024	Zn1024	Zn1024	Zn1024	Zn1024	Zn1024	Zn1024	Zn1024	Zn1024	Zn1024

Table S2. *Interactions of both isomers of compounds* **8-14** *with amino acids of porcine aminopeptidase (pAPN) taken for refinement during the IFD.*

**All**	**8R**	**8S**	**9R**	**9S**	**10R**	**10S**	**11R**	**11S**	**12R**	**12S**	**13R**	**13S**	**14R**	**14S**
**Gln206**														
**Gln208**				Gln208			Gln208					Gln208		Gln208
**Phe344**				Phe344			Phe344					Phe344	Phe344	Phe344
**Asn345**													Asn345	
**Ala346**	Ala346	Ala346	Ala346	Ala346	Ala346	Ala346	Ala346	Ala346	Ala346	Ala346	Ala346	Ala346	Ala346	Ala346
**Gly347**	Gly347	Gly347	Gly347	Gly347	Gly347	Gly347	Gly347	Gly347	Gly347	Gly347	Gly347	Gly347	Gly347	Gly347
**Ala348**	Ala348	Ala348	Ala348	Ala348	Ala348	Ala348	Ala348	Ala348	Ala348	Ala348	Ala348	Ala348	Ala348	Ala348
**Met349**	Met349	Met349	Met349	Met349	Met349	Met349	Met349	Met349	Met349	Met349	Met349	Met349	Met349	Met349
**Glu350**	Glu350	Glu350	Glu350	Glu350	Glu350	Glu350	Glu350	Glu350	Glu350	Glu350	Glu350	Glu350		Glu350
**Tyr357**					Tyr357		Tyr357		Tyr357					Tyr357
**Arg358**		Arg358				Arg358		Arg358		Arg358			Arg358	
**Asn360**													Asn360	
**Ala361**		Ala361						Ala361		Ala361			Ala361	
**Arg376**	Arg376	Arg376	Arg376		Arg376	Arg376		Arg376	Arg376	Arg376	Arg376		Arg376	
**Thr379**	Thr379		Thr379	Thr379			Thr379		Thr379			Thr379		Thr379
**Val380**	Val380	Val380	Val380	Val380	Val380	Val380	Val380	Val380	Val380	Val380	Val380	Val380	Val380	Val380
**Ile381**							Ile381							
**His383**	His383	His383	His383	His383	His383	His383	His383	His383	His383	His383	His383	His383	His383	His383
**Glu384**	Glu384	Glu384	Glu384	Glu384	Glu384	Glu384	Glu384	Glu384	Glu384	Glu384	Glu384	Glu384	Glu384	Glu384
**His387**	His387	His387	His387	His387	His387	His387	His387	His387	His387	His387	His387	His387		His387
**Glu406**	Glu406	Glu406	Glu406	Glu406	Glu406	Glu406	Glu406	Glu406	Glu406	Glu406	Glu406	Glu406		Glu406
**Ala409**		Ala409	Ala409			Ala409		Ala409	Ala409	Ala409	Ala409			
**Ser410**	Ser410	Ser410	Ser410	Ser410	Ser410	Ser410	Ser410	Ser410	Ser410	Ser410	Ser410	Ser410		Ser410
**Glu413**	Glu413	Glu413	Glu413	Glu413	Glu413	Glu413	Glu413	Glu413	Glu413	Glu413	Glu413	Glu413		Glu413
**Tyr414**									Tyr414					
**Gly433**									Gly433					
**Asp434**			Asp434						Asp434		Asp434			
**Arg437**			Arg437						Arg437					
**Phe467**	Phe467	Phe467		Phe467	Phe467	Phe467	Phe467					Phe467		Phe467
**Tyr472**	Tyr472	Tyr472	Tyr472	Tyr472	Tyr472	Tyr472	Tyr472	Tyr472	Tyr472	Tyr472	Tyr472	Tyr472	Tyr472	Tyr472
**Ser473**			Ser473						Ser473		Ser473			
**Zn1024**	Zn1024	Zn1024	Zn1024	Zn1024	Zn1024	Zn1024	Zn1024	Zn1024	Zn1024	Zn1024	Zn1024	Zn1024		Zn1024

Table S3. *Interactions of both isomers of compounds* **1-7** *with amino acids of bovine lens lerucine aminopeptidase (pAPN) taken for refinement during the IFD.*

All	1R	1S	2R	2S	3R	3S	4R	4S	5R	5S	6R	6S	7R	7S
**Lys342**	Lys342	Lys342	Lys342	Lys342	Lys342	Lys342	Lys342	Lys342	Lys342	Lys342	Lys342	Lys342	Lys342	Lys342
**Lys344**	Lys344	Lys344	Lys344		Lys344	Lys344	Lys344	Lys344	Lys344	Lys344			Lys344	Lys344
**Asp347**	Asp347	Asp347	Asp347	Asp347	Asp347	Asp347	Asp347	Asp347	Asp347	Asp347	Asp347	Asp347	Asp347	Asp347
**Gly349**	Gly349	Gly349	Gly349	Gly349	Gly349	Gly349	Gly349	Gly349	Gly349	Gly349		Gly349	Gly349	Gly349
**Lys354**	Lys354	Lys354	Lys354	Lys354	Lys354	Lys354	Lys354	Lys354	Lys354	Lys354	Lys354	Lys354	Lys354	Lys354
**Arg358**		Arg358		Arg358		Arg358		Arg358		Arg358		Arg358		Arg358
**Ser359**		Ser359		Ser359		Ser359		Ser359		Ser359		Ser359		Ser359
**Arg360**	Arg360	Arg360	Arg360	Arg360	Arg360	Arg360	Arg360	Arg360	Arg360	Arg360		Arg360	Arg360	Arg360
**Ile361**				Ile361				Ile361		Ile361		Ile361		
**Leu363**														Leu363
**Met364**	Met364	Met364	Met364	Met364	Met364	Met364	Met364	Met364	Met364	Met364	Met364	Met364	Met364	Met364
**Asn366**	Asn366	Asn366	Asn366	Asn366	Asn366	Asn366	Asn366	Asn366	Asn366	Asn366		Asn366	Asn366	Asn366
**Asp367**	Asp367	Asp367	Asp367	Asp367	Asp367	Asp367	Asp367	Asp367	Asp367	Asp367	Asp367	Asp367	Asp367	Asp367
**Asn425**														
**Asp427**	Asp427	Asp427	Asp427	Asp427	Asp427	Asp427	Asp427	Asp427	Asp427	Asp427	Asp427	Asp427	Asp427	Asp427
**Ala428**	Ala428	Ala428	Ala428		Ala428	Ala428	Ala428	Ala428	Ala428	Ala428	Ala428		Ala428	Ala428
**Glu429**	Glu429	Glu429	Glu429	Glu429	Glu429	Glu429	Glu429	Glu429	Glu429	Glu429	Glu429	Glu429	Glu429	Glu429
**Gly430**	Gly430	Gly430	Gly430	Gly430	Gly430	Gly430	Gly430	Gly430	Gly430	Gly430	Gly430	Gly430	Gly430	Gly430
**Arg431**											Arg431			
**Thr454**	Thr454	Thr454	Thr454	Thr454	Thr454	Thr454	Thr454	Thr454	Thr454	Thr454	Thr454	Thr454	Thr454	Thr454
**Leu455**	Leu455	Leu455	Leu455	Leu455	Leu455	Leu455	Leu455	Leu455	Leu455	Leu455	Leu455	Leu455	Leu455	Leu455
**Thr456**	Thr456	Thr456	Thr456	Thr456	Thr456	Thr456	Thr456	Thr456	Thr456	Thr456	Thr456	Thr456	Thr456	Thr456
**Gly457**	Gly457	Gly457	Gly457	Gly457	Gly457	Gly457	Gly457	Gly457	Gly457	Gly457	Gly457	Gly457	Gly457	Gly457
**Ala458**				Ala458				Ala458		Ala458	Ala458	Ala458		
**Ile459**														
**Met460**	Met460	Met460	Met460		Met460		Met460		Met460			Met460	Met460	
**Thr515**														
**Gly518**														
**Gly521**														
**Asp543**											Asp543			
**Ala545**	Ala545	Ala545	Ala545	Ala545	Ala545	Ala545	Ala545	Ala545	Ala545	Ala545	Ala545	Ala545	Ala545	Ala545
**Gly546**														
**Val548**														
**Trp549**														
**Mg601**	Mg601	Mg601	Mg601	Mg601	Mg601	Mg601	Mg601	Mg601	Mg601	Mg601	Mg601	Mg601	Mg601	Mg601
**Mg602**	Mg602	Mg602	Mg602	Mg602	Mg602	Mg602	Mg602	Mg602	Mg602	Mg602	Mg602	Mg602	Mg602	Mg602

Table S4. *Interactions of both isomers of compounds* **8-14** *with amino acids of bovine lens lerucine aminopeptidase (pAPN) taken for refinement during the IFD.*

All	8R	8S	9R	9S	10R	10S	11R	11S	12R	12S	13R	13S	14R	14S
**Lys342**	Lys342	Lys342	Lys342	Lys342	Lys342	Lys342	Lys342	Lys342	Lys342	Lys342	Lys342	Lys342	Lys342	Lys342
**Lys344**	Lys344	Lys344	Lys344	Lys344				Lys344		Lys344	Lys344	Lys344	Lys344	Lys344
**Asp347**	Asp347	Asp347	Asp347	Asp347	Asp347	Asp347	Asp347	Asp347	Asp347	Asp347	Asp347	Asp347	Asp347	Asp347
**Gly349**	Gly349		Gly349	Gly349				Gly349		Gly349	Gly349		Gly349	
**Lys354**	Lys354	Lys354	Lys354	Lys354	Lys354	Lys354	Lys354	Lys354	Lys354	Lys354	Lys354	Lys354	Lys354	Lys354
**Arg358**				Arg358				Arg358		Arg358			Arg358	
**Ser359**		Ser359		Ser359		Ser359		Ser359		Ser359	Ser359		Ser359	Ser359
**Arg360**	Arg360	Arg360	Arg360	Arg360		Arg360		Arg360		Arg360	Arg360		Arg360	Arg360
**Ile361**				Ile361				Ile361					Ile361	
**Leu363**		Leu363				Leu363		Leu363						Leu363
**Met364**	Met364	Met364	Met364	Met364	Met364	Met364	Met364	Met364	Met364	Met364	Met364	Met364	Met364	Met364
**Asn366**	Asn366	Asn366	Asn366	Asn366		Asn366		Asn366		Asn366			Asn366	Asn366
**Asp367**	Asp367	Asp367	Asp367	Asp367	Asp367	Asp367	Asp367	Asp367	Asp367	Asp367	Asp367	Asp367	Asp367	Asp367
**Asn425**												Asn425		
**Asp427**	Asp427	Asp427	Asp427	Asp427	Asp427	Asp427	Asp427	Asp427	Asp427	Asp427	Asp427	Asp427	Asp427	Asp427
**Ala428**	Ala428	Ala428	Ala428	Ala428	Ala428	Ala428	Ala428	Ala428	Ala428	Ala428	Ala428	Ala428	Ala428	Ala428
**Glu429**	Glu429	Glu429	Glu429	Glu429	Glu429	Glu429	Glu429	Glu429	Glu429	Glu429	Glu429	Glu429	Glu429	Glu429
**Gly430**	Gly430	Gly430	Gly430	Gly430	Gly430	Gly430	Gly430	Gly430	Gly430	Gly430	Gly430	Gly430	Gly430	Gly430
**Arg431**		Arg431			Arg431	Arg431	Arg431		Arg431		Arg431	Arg431		Arg431
**Thr454**	Thr454	Thr454	Thr454	Thr454	Thr454	Thr454	Thr454	Thr454	Thr454	Thr454	Thr454	Thr454	Thr454	Thr454
**Leu455**	Leu455	Leu455	Leu455	Leu455	Leu455	Leu455	Leu455	Leu455	Leu455	Leu455	Leu455	Leu455	Leu455	Leu455
**Thr456**	Thr456	Thr456	Thr456	Thr456	Thr456	Thr456	Thr456	Thr456	Thr456	Thr456	Thr456	Thr456	Thr456	Thr456
**Gly457**	Gly457	Gly457	Gly457	Gly457	Gly457	Gly457	Gly457	Gly457	Gly457	Gly457	Gly457	Gly457	Gly457	Gly457
**Ala458**		Ala458		Ala458	Ala458	Ala458	Ala458	Ala458	Ala458		Ala458	Ala458	Ala458	Ala458
**Ile459**	Ile459													
**Met460**	Met460	Met460	Met460			Met460		Met460		Met460				Met460
**Thr515**							Thr515							
**Gly518**							Gly518		Gly518					
**Gly521**							Gly521							
**Asp543**					Asp543		Asp543		Asp543					
**Ala545**	Ala545	Ala545	Ala545	Ala545	Ala545	Ala545	Ala545	Ala545	Ala545	Ala545	Ala545		Ala545	Ala545
**Gly546**														Gly546
**Val548**		Val548												Val548
**Trp549**														Trp549
**Mg601**	Mg601	Mg601	Mg601	Mg601	Mg601	Mg601	Mg601	Mg601	Mg601	Mg601	Mg601	Mg601	Mg601	Mg601
**Mg602**	Mg602	Mg602	Mg602	Mg602	Mg602	Mg602	Mg602	Mg602	Mg602	Mg602	Mg602	Mg602	Mg602	Mg602

Table S5. *Interactions of both isomers of compounds* ***1-7*** *with amino acids of tomato acidic lerucine aminopeptidase (pAPN) taken for refinement during the IFD.*

**All**	**1R**	**1S**	**2R**	**2S**	**3R**	**3S**	**4R**	**4S**	**5R**	**5S**	**6R**	**6S**	**7R**	**7S**
**Lys342**	Lys342	Lys342	Lys342	Lys342	Lys342	Lys342	Lys342	Lys342	Lys342	Lys342	Lys342	Lys342	Lys342	Lys342
**Lys344**	Lys344	Lys344	Lys344		Lys344	Lys344	Lys344	Lys344	Lys344	Lys344			Lys344	Lys344
**Asp347**	Asp347	Asp347	Asp347	Asp347	Asp347	Asp347	Asp347	Asp347	Asp347	Asp347	Asp347	Asp347	Asp347	Asp347
**Gly349**	Gly349	Gly349	Gly349	Gly349	Gly349	Gly349	Gly349	Gly349	Gly349	Gly349		Gly349	Gly349	Gly349
**Lys354**	Lys354	Lys354	Lys354	Lys354	Lys354	Lys354	Lys354	Lys354	Lys354	Lys354	Lys354	Lys354	Lys354	Lys354
**Arg358**		Arg358		Arg358		Arg358		Arg358		Arg358		Arg358		Arg358
**Ser359**		Ser359		Ser359		Ser359		Ser359		Ser359		Ser359		Ser359
**Arg360**	Arg360	Arg360	Arg360	Arg360	Arg360	Arg360	Arg360	Arg360	Arg360	Arg360		Arg360	Arg360	Arg360
**Ile361**				Ile361				Ile361		Ile361		Ile361		
**Leu363**														Leu363
**Met364**	Met364	Met364	Met364	Met364	Met364	Met364	Met364	Met364	Met364	Met364	Met364	Met364	Met364	Met364
**Asn366**	Asn366	Asn366	Asn366	Asn366	Asn366	Asn366	Asn366	Asn366	Asn366	Asn366		Asn366	Asn366	Asn366
**Asp367**	Asp367	Asp367	Asp367	Asp367	Asp367	Asp367	Asp367	Asp367	Asp367	Asp367	Asp367	Asp367	Asp367	Asp367
**Asn425**														
**Asp427**	Asp427	Asp427	Asp427	Asp427	Asp427	Asp427	Asp427	Asp427	Asp427	Asp427	Asp427	Asp427	Asp427	Asp427
**Ala428**	Ala428	Ala428	Ala428		Ala428	Ala428	Ala428	Ala428	Ala428	Ala428	Ala428		Ala428	Ala428
**Glu429**	Glu429	Glu429	Glu429	Glu429	Glu429	Glu429	Glu429	Glu429	Glu429	Glu429	Glu429	Glu429	Glu429	Glu429
**Gly430**	Gly430	Gly430	Gly430	Gly430	Gly430	Gly430	Gly430	Gly430	Gly430	Gly430	Gly430	Gly430	Gly430	Gly430
**Arg431**											Arg431			
**Thr454**	Thr454	Thr454	Thr454	Thr454	Thr454	Thr454	Thr454	Thr454	Thr454	Thr454	Thr454	Thr454	Thr454	Thr454
**Leu455**	Leu455	Leu455	Leu455	Leu455	Leu455	Leu455	Leu455	Leu455	Leu455	Leu455	Leu455	Leu455	Leu455	Leu455
**Thr456**	Thr456	Thr456	Thr456	Thr456	Thr456	Thr456	Thr456	Thr456	Thr456	Thr456	Thr456	Thr456	Thr456	Thr456
**Gly457**	Gly457	Gly457	Gly457	Gly457	Gly457	Gly457	Gly457	Gly457	Gly457	Gly457	Gly457	Gly457	Gly457	Gly457
**Ala458**				Ala458				Ala458		Ala458	Ala458	Ala458		
**Ile459**														
**Met460**	Met460	Met460	Met460		Met460		Met460		Met460			Met460	Met460	
**Thr515**														
**Gly518**														
**Gly521**														
**Asp543**											Asp543			
**Ala545**	Ala545	Ala545	Ala545	Ala545	Ala545	Ala545	Ala545	Ala545	Ala545	Ala545	Ala545	Ala545	Ala545	Ala545
**Gly546**														
**Val548**														
**Trp549**														
**Mg601**	Mg601	Mg601	Mg601	Mg601	Mg601	Mg601	Mg601	Mg601	Mg601	Mg601	Mg601	Mg601	Mg601	Mg601
**Mg602**	Mg602	Mg602	Mg602	Mg602	Mg602	Mg602	Mg602	Mg602	Mg602	Mg602	Mg602	Mg602	Mg602	Mg602

Table S6. *Interactions of both isomers of compounds***1-7** *with amino acids of tomato acidic lerucine aminopeptidase (pAPN) taken for refinement during the IFD*

All	8R	8S	9R	9S	10R	10S	11R	11S	12R	12S	13R	13S	14R	14S
**Lys342**	Lys342	Lys342	Lys342	Lys342	Lys342	Lys342	Lys342	Lys342	Lys342	Lys342	Lys342	Lys342	Lys342	Lys342
**Lys344**	Lys344	Lys344	Lys344	Lys344				Lys344		Lys344	Lys344	Lys344	Lys344	Lys344
**Asp347**	Asp347	Asp347	Asp347	Asp347	Asp347	Asp347	Asp347	Asp347	Asp347	Asp347	Asp347	Asp347	Asp347	Asp347
**Gly349**	Gly349		Gly349	Gly349				Gly349		Gly349	Gly349		Gly349	
**Lys354**	Lys354	Lys354	Lys354	Lys354	Lys354	Lys354	Lys354	Lys354	Lys354	Lys354	Lys354	Lys354	Lys354	Lys354
**Arg358**				Arg358				Arg358		Arg358			Arg358	
**Ser359**		Ser359		Ser359		Ser359		Ser359		Ser359	Ser359		Ser359	Ser359
**Arg360**	Arg360	Arg360	Arg360	Arg360		Arg360		Arg360		Arg360	Arg360		Arg360	Arg360
**Ile361**				Ile361				Ile361					Ile361	
**Leu363**		Leu363				Leu363		Leu363						Leu363
**Met364**	Met364	Met364	Met364	Met364	Met364	Met364	Met364	Met364	Met364	Met364	Met364	Met364	Met364	Met364
**Asn366**	Asn366	Asn366	Asn366	Asn366		Asn366		Asn366		Asn366			Asn366	Asn366
**Asp367**	Asp367	Asp367	Asp367	Asp367	Asp367	Asp367	Asp367	Asp367	Asp367	Asp367	Asp367	Asp367	Asp367	Asp367
**Asn425**												Asn425		
**Asp427**	Asp427	Asp427	Asp427	Asp427	Asp427	Asp427	Asp427	Asp427	Asp427	Asp427	Asp427	Asp427	Asp427	Asp427
**Ala428**	Ala428	Ala428	Ala428	Ala428	Ala428	Ala428	Ala428	Ala428	Ala428	Ala428	Ala428	Ala428	Ala428	Ala428
**Glu429**	Glu429	Glu429	Glu429	Glu429	Glu429	Glu429	Glu429	Glu429	Glu429	Glu429	Glu429	Glu429	Glu429	Glu429
**Gly430**	Gly430	Gly430	Gly430	Gly430	Gly430	Gly430	Gly430	Gly430	Gly430	Gly430	Gly430	Gly430	Gly430	Gly430
**Arg431**		Arg431			Arg431	Arg431	Arg431		Arg431		Arg431	Arg431		Arg431
**Thr454**	Thr454	Thr454	Thr454	Thr454	Thr454	Thr454	Thr454	Thr454	Thr454	Thr454	Thr454	Thr454	Thr454	Thr454
**Leu455**	Leu455	Leu455	Leu455	Leu455	Leu455	Leu455	Leu455	Leu455	Leu455	Leu455	Leu455	Leu455	Leu455	Leu455
**Thr456**	Thr456	Thr456	Thr456	Thr456	Thr456	Thr456	Thr456	Thr456	Thr456	Thr456	Thr456	Thr456	Thr456	Thr456
**Gly457**	Gly457	Gly457	Gly457	Gly457	Gly457	Gly457	Gly457	Gly457	Gly457	Gly457	Gly457	Gly457	Gly457	Gly457
**Ala458**		Ala458		Ala458	Ala458	Ala458	Ala458	Ala458	Ala458		Ala458	Ala458	Ala458	Ala458
**Ile459**	Ile459													
**Met460**	Met460	Met460	Met460			Met460		Met460		Met460				Met460
**Thr515**							Thr515							
**Gly518**							Gly518		Gly518					
**Gly521**							Gly521							
**Asp543**					Asp543		Asp543		Asp543					
**Ala545**	Ala545	Ala545	Ala545	Ala545	Ala545	Ala545	Ala545	Ala545	Ala545	Ala545	Ala545		Ala545	Ala545
**Gly546**														Gly546
**Val548**		Val548												Val548
**Trp549**														Trp549
**Mg601**	Mg601	Mg601	Mg601	Mg601	Mg601	Mg601	Mg601	Mg601	Mg601	Mg601	Mg601	Mg601	Mg601	Mg601
**Mg602**	Mg602	Mg602	Mg602	Mg602	Mg602	Mg602	Mg602	Mg602	Mg602	Mg602	Mg602	Mg602	Mg602	Mg602

Enzyme assays

The progress of the enzymatic reactions was monitored spectrophotometrically (UV-VIS Spectrophotometer JASCO V-730) by following the change in absorbance at 405 nm (formation of *p*-nitroanilide). The extinction coefficient for *L*-Leu-pNa was 9620 $M^{-1}cm^{-1}$.

The kinetic parameters (K_M and V_{max}) for aminopeptidase from barley seeds were determined by using the Lineweaver–Burk (L-B) weighted regression method. The measured K_M value was 0.21 mM ± 0.14 and was used to determine the inhibitory constants of the all evaluated inhibitors.

Table S7. The kinetic parameters characterizing activity of the isolated aminopeptidase from barley seeds.

Metoda	K_M	S_{KM}	V_{max}	S_{Vmax}	R^2	a	b
L-B unweighted	0,218533	0,008905	0,009876	0,000186102	0,981321	1,001484	-2,7E-06
L-B weighted	0,210435	0,014394	0,009782	0,000190483	0,981347	0,985662	0,000112

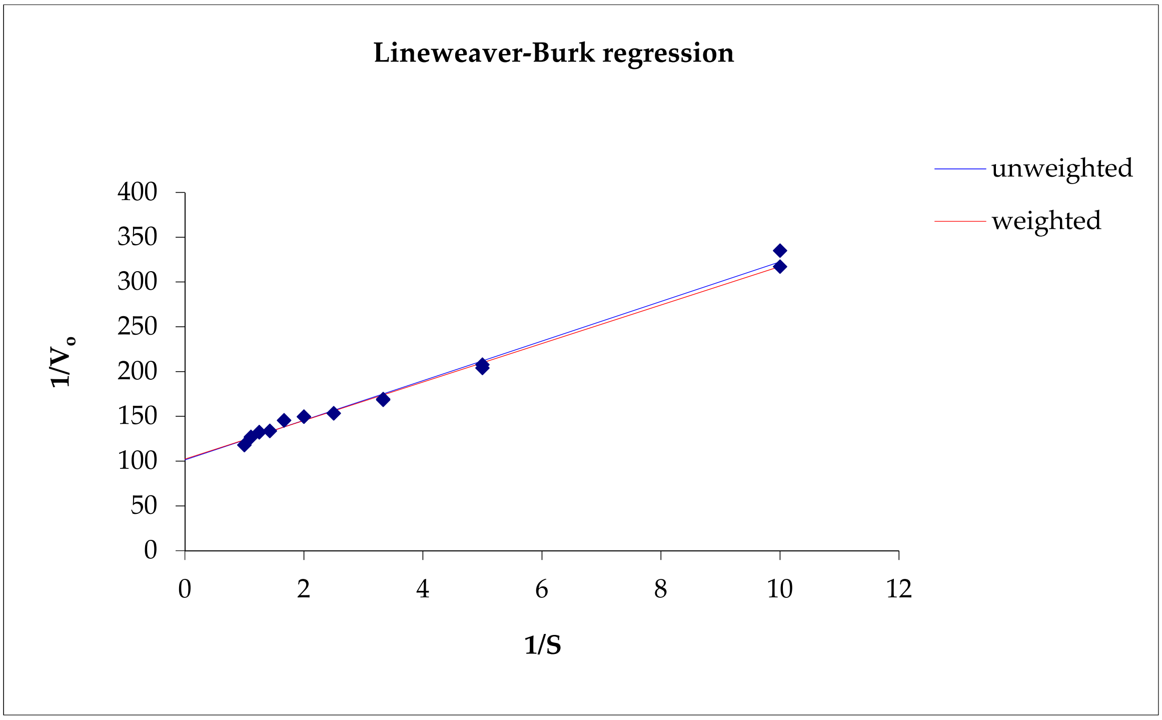

Plot S1. Graphical determination of the K_M value for aminopeptidase from barley seeds by using Lineweaver-Burk: unweighted (blue line) and weighted (red line) regression method.

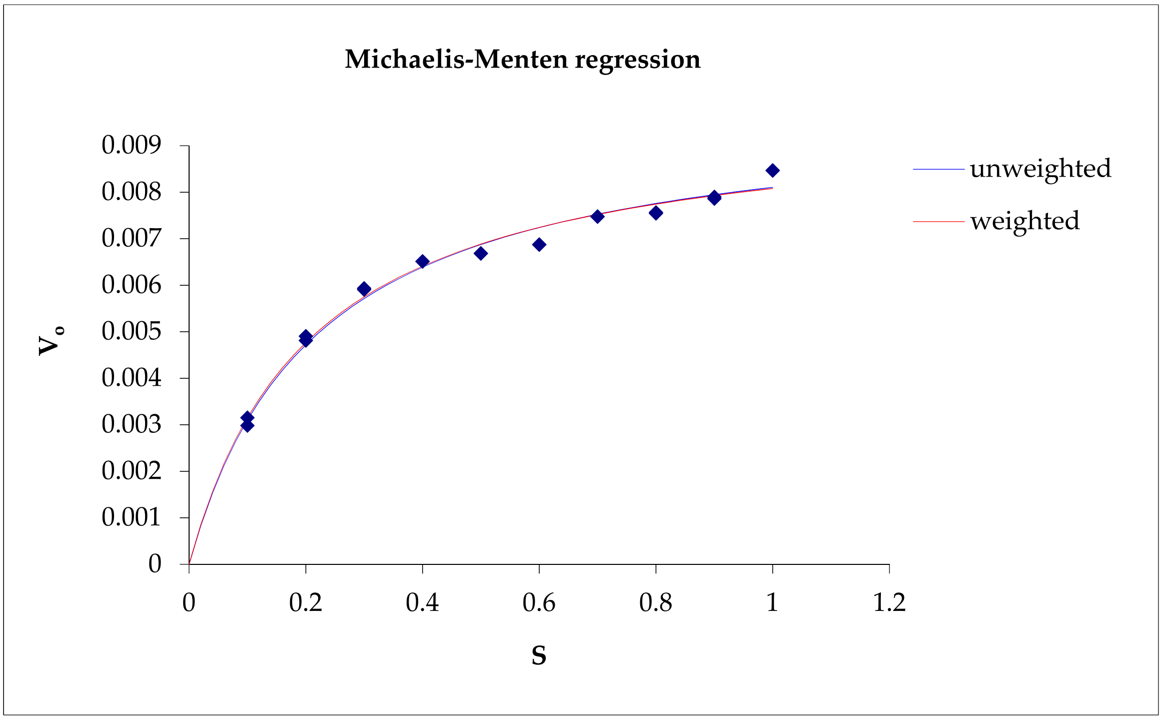

Plot S2. Graphical determination of the K_M value for aminopeptidase from barley seeds by using Michaelis-Menten: unweighted (blue line) and weighted (red line) non-linear regression method.

The value of K_M for porcine aminopeptidase (pAPN) was 0.52mM and was determined as described above.
